# m6A methylation mediates LHPP acetylation as a tumour aerobic glycolysis suppressor to improve the prognosis of gastric cancer

**DOI:** 10.1038/s41419-022-04859-w

**Published:** 2022-05-14

**Authors:** Jian-Xian Lin, Ning-Zi Lian, You-Xin Gao, Qiao-Ling Zheng, Ying-Hong Yang, Yu-Bin Ma, Zhi-Song Xiu, Qing-Zhu Qiu, Hua-Gen Wang, Chao-Hui Zheng, Ping Li, Jian-Wei Xie, Jun Lu, Qi-Yue Chen, Long-Long Cao, Mi Lin, Jia-Bin Wang, Chang-Ming Huang

**Affiliations:** 1 Department of Gastric Surgery, Fujian Medical University Union Hospital, Fuzhou, China; 2 Key Laboratory of Ministry of Education of Gastrointestinal Cancer, Fujian Medical University, Fuzhou, China; 3 Fujian Key Laboratory of Tumor Microbiology, Fujian Medical University, Fuzhou, China; 4 Department of Gynecology, Fujian Obstetrics and Gynecology Hospital, Fuzhou, China; 5 Department of Pathology, Fujian Medical University Union Hospital, Fuzhou, China; 6 Department of Gastrointestinal Surgery, the Affiliated Hospital of Qinghai University, Xining, China; 7 Public Health School of Fujian Medical University, Fuzhou, China

**Keywords:** Gastric cancer, Predictive markers

## Abstract

LHPP, a histidine phosphatase, has been implicated in tumour progression. However, its role, underlying mechanisms, and prognostic significance in human gastric cancer (GC) are elusive. Here, we obtained GC tissues and corresponding normal tissues from 48 patients and identified LHPP as a downregulated gene via RNA-seq. qRT-PCR and western blotting were applied to examine LHPP levels in normal and GC tissues. The prognostic value of LHPP was elucidated using tissue microarray and IHC analyses in two independent GC cohorts. The functional roles and mechanistic insights of LHPP in GC growth and metastasis were evaluated in vitro and in vivo. The results showed that LHPP expression was significantly decreased in GC tissues at both the mRNA and protein levels. Multivariate Cox regression analysis revealed that LHPP was an independent prognostic factor and effective predictor in patients with GC. The low expression of LHPP was significantly related to the poor prognosis and chemotherapy sensitivity of gastric cancer patients. Moreover, elevated LHPP expression effectively suppressed GC growth and metastasis in vitro and in vivo. Mechanistically, the m6A modification of LHPP mRNA by METTL14 represses its expression; LHPP inhibits the phosphorylation of GSK3b through acetylation and mediates HIF1A to inhibit glycolysis, proliferation, invasion and metastasis of gastric cancer cells. Together, our findings suggest that LHPP is regulated by m6A methylation and regulates the metabolism of GC by changing the acetylation level. Thus, LHPP is a potential predictive biomarker and therapeutic target for GC.

## Background

Gastric cancer (GC) is the third most common tumour-related disease worldwide [1, 2]. Cancer cells need to effectively coordinate glycolysis and glutamate decomposition to meet their bioenergy and biosynthesis requirements for proliferation and survival [3, 4]. However, the metabolic adaptations of GC and the mechanisms of tumour proliferation, invasion and metastasis have not been fully elucidated, and there is a lack of specific and efficient intervention methods in clinical practice [5, 6]. Therefore, identifying molecular markers and their mechanism of action which inhibits the metabolic adaptation, invasion, and metastasis of GC can provide new targeted therapies which have important theoretical and clinical significance.

Phospholysine phosphohistidine inorganic pyrophosphate phosphatase (LHPP) is a histidine phosphatase, whose role is opposite to that of histidine kinase, which can remove protein and histamine, acid-linked phosphate groups [7]. The human *LHPP* gene (NM_022126) is located on chromosome 10 (10q26.13). It is an acidic protein containing 270 amino acids and is a non-transmembrane protein, which is mainly located in the cytoplasm and is expressed in most tissues [8–10]. Previous studies have found that *LHPP* is a genetic marker of alcohol dependence and severe depression and is related to mitochondrial dysfunction and chronic oxidative stress [10, 11]. Two genome-wide association studies have found that *LHPP* is a susceptibility gene for primary open-angle glaucoma, oropharyngeal cancer, and acute lymph node cell leukaemia [12, 13]. A study by Hindupur et al. reported that overexpression of LHPP can inhibit liver tumour formation, maintain liver function, and is related to patient prognosis [14]. However, whether and how LHPP regulates metabolic adaptation in GC remains unclear.

By investigating the upstream regulatory mechanisms of LHPP, we found that its mRNA level may be related to N6-methyladenosine (m6A) methylation modification. m6A methylation is the most prevalent internal chemical modification of eukaryotic mRNAs [15]. Previous studies have reported that the effects of m6A modification include regulating mRNA stability, splicing, and translation [16]. Methyltransferase-like 14 (METTL14) is a catalytic enzyme, which promotes m6A modification of mRNA [17]. However, the mechanism by which METTL14 mediates the m6A modification of LHPP to affect GC progression has not yet been reported.

Bioinformatics analyses of public databases revealed that LHPP may affect tumour glycolysis through acetylation. The prominent feature of tumour cell metabolic adaptation is the Warburg effect, that is, regardless of the oxygen state, many cancer cells rely on high glucose uptake rates and convert most of the glucose into lactic acid through glycolysis rather than catabolising glucose through oxidative phosphorylation [18]. The Warburg effect may be caused by the hypoxic microenvironment of the tumour, abnormal signalling pathways, abnormal activation of oncogenes, glucose transporters, or the overexpression of enzymes in the glycolytic pathway [19].

In this study, we investigated the clinical significance and regulatory mechanism of LHPP in GC.

## Materials and methods

### Patients and gastric tissue sample

The study was primarily based on two independent patient cohorts. Cohort 1 included 349 gastric cancer tissues collected from January 2010 to April 2014 at Fujian Medical University Union Hospital. Gastric tissue specimens included tumour tissues of the stomach and adjacent non-tumour tissues. Cohort 2 included 93 gastric cancer tissues collected from January 2010 to April 2014 at Qinghai University Hospital for external validation. The inclusion criteria were as follows: (a) histological identification of gastric cancer; (b) availability of follow-up data and clinicopathological characteristics; (c) TNM staging of gastric cancer tumours according to the 2010 International Union Against Cancer (UICC) guidelines. The exclusion criteria were as follows: (1) patients with no formalin-fixed paraffin-embedded (FFPE) tumour sample (CT and IM) from initial diagnosis; (2) patients who received chemotherapy or radiotherapy before surgery. All participating patients with advanced GC routinely received fluorine-based chemotherapy (Table 1).

**Table 1:** Relationship between LHPP expression and baseline characteristics of patients.

Variables	Internal set			External set		
	LHPP^low^	LHPP^high^	*P*	LHPP^low^	LHPP^high^	*P*
**All patients**	214	135		53	40	
**Gender**			0.581			0.816
Female	53	35		15	12	
Male	161	100		38	28	
**Age at surgery(yr)**			0.025			0.454
<65	126	77		39	33	
≥65	88	58		14	7	
**BMI**			0.458			0.555
<25	186	113		44	37	
≥25	28	22		9	3	
**TNM stage**			0.000			0.001
I	24	23		11	9	
II	74	43		19	15	
III	116	69		23	16	
Chemotherapy*			0.004			0.013
No	100	77		31	16	
Yes	114	58		22	24	
**Tumour size (mm)**			0.000			0.030
≤40	77	57		26	25	
>40	137	78		27	15	
**Resection type**			0.336			0.167
Part gastrectomy	98	58		40	36	
Total gastrectomy	116	77		13	4	
**Pathological type**			0.973			0.175
Adenocarcinoma	176	113		44	38	
mix	32	12		8	1	
non Adenocarcinoma	6	10		1	1	

*P* < 0.05 marked in bold font shows statistical significance.

*Adjuvant chemotherapy after surgery, no radiotherapy was administered to anyone of the patients enrolled.

### Construction of tissue microarray (TMA)

From January 2010 to April 2014, a total of 123 gastric cancer tissue samples were selected. Briefly, the pathologist examined all gastric cancer tissues, marked the paraffin blocks based on the tumour position of the HE stained section and immunohistochemical slides, and selected more areas of the tumour tissue without the representation of necrosis and haemorrhagic material Area to prepare tissue chips for experiments. Mix paraffin wax and an equal amount of beeswax to make two blank wax blocks. Create a puncture hole with a diameter of 1 mm in blank paraffin to separate the two holes, and perform 80 punches. For each sample, a 1.5 mm core was punched from the donor block using a tissue microarray instrument. Use a tissue analyzer to sample the tumour-marked wax block, put the sampled tissue into the corresponding channel of the blank wax block, and transfer the determined array position to the recipient paraffin block. Several serial sections (4 μm thick) were cut from all TMAs, and one section of each TMA was stained with hematoxylin-eosin to ensure that the TMA was constructed correctly. The intratumor dot was derived from the centre of the tumour, while the peritumor dot was punched out from the area ≥2 cm from the tumour margin. The prepared TMA slides are used for immunohistochemistry (IHC).

### Follow-up

All patients were systematically followed up by trained doctors who abided by the institutional follow-up protocol; options for follow-up included outpatient services, letters, telephone, mail or visits. Follow-up was conducted every 3–6 months for the first 2 years, every 6–12 months for the 3–5 years, and annually thereafter. Survival time was defined as the time from the date of surgery until the date of the last follow-up or death. All 565 patients involved in the IHC analysis completed the follow-up.

### Immunohistochemistry (IHC)

The serial sections of the FFPE sample were 4 μm and mounted on a glass slide for IHC analysis. The sections were deparaffinized with xylene and rehydrated with alcohol. We blocked the endogenous peroxidase by immersing the slices in a 3% H_2_O_2_ aqueous solution for 10 min and microwaved them in 0.01 mol/L sodium citrate buffer (pH 6.0) for 10 min for antigen retrieval. The slides were then washed in phosphate-buffered saline (PBS) and then incubated with 10% normal goat serum (Zhongshan Biotechnology Co., Ltd., China) to eliminate nonspecific reactions. Subsequently, the primary antibody was incubated with the antibody overnight at 4 °C. The treatment of the negative control is the same, but the primary antibody is omitted. After rinsing three times with PBS, dilute the slide and secondary antibody for 30 min at room temperature, and develop with diaminobenzidine (DAB) solution. Finally, the slides were counter-stained with hematoxylin, dehydrated and fixed with a cover glass and neutral resin.

We performed LHPP (NBP1-83273, Novus, 1:300), METTL14 (ab220030, Abcam, 1:1000), HIF-1α (ab243861, Abcam, 1:500), GSK-3β (phospho S9) (ab75814, Abcam, 1:200) immunohistochemical staining on the tumour tissue of gastric cancer patients. The staining intensity and average percentage of positive cells in five randomly selected regions were evaluated to represent the protein expression level. The scoring criteria are as follows: staining intensity is divided into 0 (negative staining), 1 (weak staining, light yellow), 2 (medium staining, yellow-brown) or 3 (strong staining, brown), and positive staining of tumour cells The proportion is divided into 0 (≤5% positive cells), 1 (6–25% positive cells), 2 (26–50% positive cells) or 3 (≥51% positive cells) cells). The final expression is calculated by multiplying the staining intensity score by the proportional staining score (total 0 to 9). Patients with final scores of 0, 1, 2 and 3 were classified as low expression group, and patients with scores of 4, 6 and 9 were classified as high expression group.

The IHC results were evaluated by two independent gastroenterology pathologists who were blinded to the clinical data prognosis of the patients. Approximately 90% of the scoring results are the same. When the scores of the two independent pathologists diverged, another pathologist checked the results again and chose one of the scores of the first two doctors, or the three pathologists discussed the decision together.

### Gene set enrichment analysis

Gene set enrichment analysis (GSEA) performed by the Molecular Signature Database (MSigDB) was used to identify the pathways and functions that were significantly enriched in LHPP low tumour samples. If a gene set had a positive enrichment score, the majority of its members had higher expression accompanied by a higher risk score, and the set was termed ‘enriched’.

### Statistical analysis

All data were processed using SPSS 25.0 (SPSS Inc. Chicago, IL) and R software (version 4.0.0). Data were presented as the mean ± SD and analyzed using Student’s *t*-test or one-way ANOVA. For results with less than ten samples per group, we performed the Wilcoxon test. We performed repeat measure ANOVA in data involving multiple time points. The Kaplan–Meier method was used to estimate median survival. We defined the survival time of patients who were lost to follow-up as the time from surgery to the last follow-up time, and the survival time of patients who were still alive in the end was defined as the time from surgery to the database deadline. A two-tailed *P* values <0.05 were considered significant difference.

### RNA-sequencing analysis

Total RNA extraction was performed with TRIzol Reagent (Invitrogen, Carlsbad, CA, USA). RNA-sequencing analysis was performed at KangChen Bio-tech Inc. (Shanghai, China). A total of 48 pairs of cancer tissue samples and paracancerous samples (5 cm away from the tumour tissues) were included and sequenced, and their cancer tissue and paracancerous tissue were completely matched. We identified differentially expressed genes (DEGs) between the two groups. According to the 'LIMMA' software package, genes with a threshold *P* value <0.05 and Log2FC ≥ 0.585 were selected as the differential genes under study in the two groups, respectively.

### Establishment of cell lines

Overexpression and knockdown lentiviruses for LHPP (NM_022126.4), as well as control lentivirus, were purchased from GeneChem Corporation (Shanghai, China). Transfection was performed according to the manufacturer’s instructions. Puromycin (2 μg/ml, Sigma) was used to select stable clones for at least 1 week. At the indicated time points, the cells were harvested for mRNA and protein analysis as well as for other assays.

### RNA immunoprecipitation (RIP)

LHPP m6A immunoprecipitation was performed using a Magna MeRIP m6A Kit (17–10499, Merck Millipore, USA) according to the manufacturer’s protocol, and the immunoprecipitated RNA extracts were reverse transcribed and analyzed by qRT-PCR.

### Human phosphokinase array

The relative levels of protein phosphorylation were tested using the Human PhosphoKinase Array Kit (ARY003B, R&D Systems, Inc. USA and Canada) according to the manufacturer’s protocol. An equal amount of protein (600 mg) was extracted from stable cells (Overexpression LHPP HGC-27 and Control HGC-27) and used to compare the kinase activity with or without LHPP overexpression.

### Tumour formation and metastasis assays

All male BALB/c nude mice (4–5 weeks old) used in our study were purchased from Beijing Vital River Laboratory Animal Technology Co., Ltd. A total of 5 × 10^6^ stably transfected MGC-803 cells were subcutaneously injected into the right axillary fossa of nude mice. Tumour volume was measured every 3 days and calculated with the following formula: V = (L × W^2^)/2 cm^2^ (V, tumour volume; L, length; W, width). The mice were sacrificed at 3–4 weeks after injection, and the tumours were weighed. For the lung or liver metastasis model, 5 × 10^6^ stably transfected MGC-803 cells were injected into the tail veins or the spleen of nude mice. Forty-five days later, the mice were sacrificed, and the lungs or the livers were dissected to examine the histopathological metastatic loci. The peritoneal dissemination ability of GC cells was evaluated via intraperitoneal injection. A total of 5 × 10^6^ stably transfected MGC-803 cells in 500 μl of PBS were injected into the peritoneal cavity of BALB/c nude mice. Mice were carefully monitored until they were killed at 4 weeks, at which point peritoneal metastases were examined and recorded. All animal experiments were performed according to the Animal Protection Committee of Fujian Medical University (Fuzhou, China) and approved by the Ethics Committee of Fujian Medical University/Laboratory Animal Centre (Fuzhou, China).

### Western blot assay

Samples and cells were collected for Western blotting as previously described. Western blot analysis was performed using the following antibodies: LHPP (NBP1-83273, Novus, 0.2 ug/ml), METTL14 (ab220030, Abcam, 1:1000), HIF-1α (ab243861, Abcam, 1:1000), β-ACTIN (ab8226, Abcam, 1:2000), Acetyl Lysine (ab190479, Abcam, 1:1000), P300 (ab10485, Abcam, 1:5000), TIP60 (ab151432, Abcam, 1:1000), GCN5 (ab282176, Abcam, 1:1000), PCAF (ab176316, Abcam, 1:1000), GLUT1 (ab115730, Abcam, 1:5000), c-Myc (ab32072, Abcam, 1:1000), PKM2 (ab137852, Abcam, 1:1000), ALDOLASE (ab252953, Abcam, 1:1000), ENOLASE1 (H00002023-M01, Novus, 1:500), GLS1 (H00002744-M01, Novus, 1:500), GSK-3β (phospho S9) (ab75814, Abcam, 1:5000), GSK-3β (ab32391, Abcam, 1:5000), β-CATENIN (phospho S37) (ab75777, Abcam, 1:500).

## Results

### Decreased LHPP expression correlated with poor prognosis in patients with GC

**Fig. 1:**
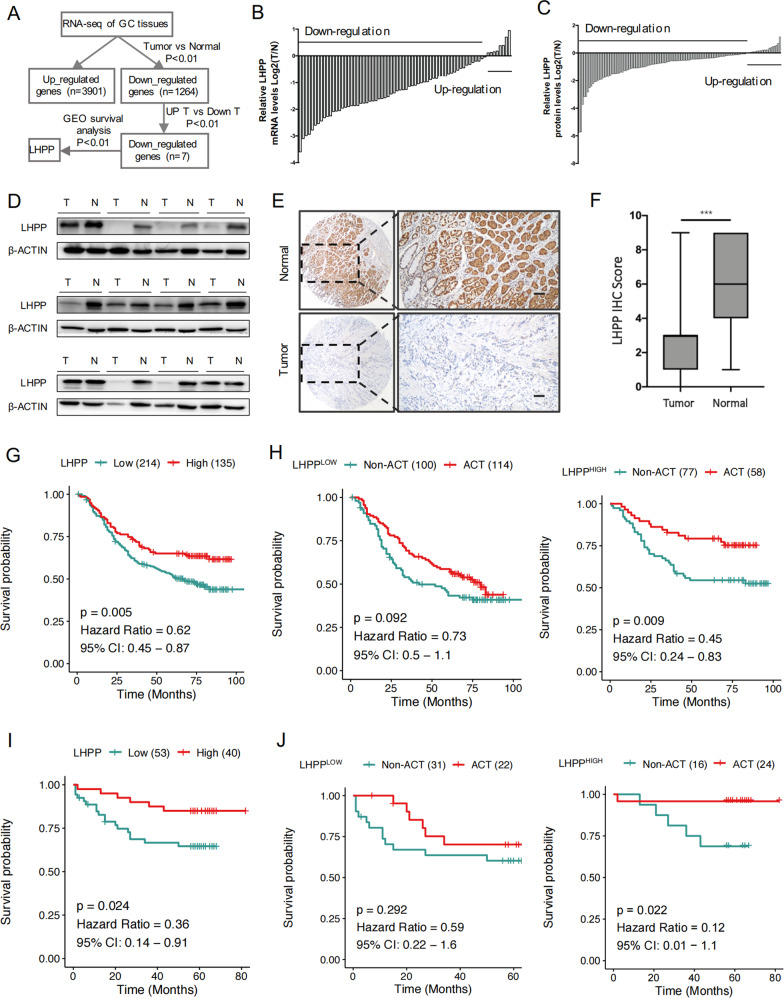
Expression and prognostic value of LHPP in GC. **A** Flowchart of the screening process of candidate genes. **B** mRNA levels of LHPP in gastric tumours and adjacent normal tissues were measured by qRT-PCR. **C** LHPP protein levels in gastric tumours and adjacent normal tissues were measured by western blot. **D** Representative images of LHPP protein levels in gastric tumours and adjacent normal tissues. **E** Expression of LHPP in 123 paraffin-embedded specimens from the internal cohort was determined by TMA-based IHC staining. Scale bars = 200 μm. **F** LHPP IHC score of gastric tumours and adjacent normal tissues in Fig. 1F. Data were presented as the mean ± SD and were analysed using Student’s *t*-test. **G** Kaplan–Meier analyses the correlations between LHPP expression and overall survival in the internal cohort. **H** Kaplan–Meier analyses the correlations between ACT and overall survival in the internal cohort stratified by LHPP expression. **I** Kaplan–Meier analyses the correlations between LHPP expression and overall survival in the external cohort. **J** Kaplan–Meier analyses the correlations between ACT and overall survival in the external cohort stratified by LHPP expression. *P* values for all survival analyses were calculated using the log-rank test. ^***^P < 0.001, GC gastric cancer, qRT-PCR quantitative reverse transcription-polymerase chain reaction, TMA tissue microarray, IHC immunohistochemistry, ACT adjuvant chemotherapy, Non-ACT not receiving adjuvant chemotherapy.

To identify genes with prognostic value in GC, we performed transcriptome sequencing on the tumour and adjacent tissues of patients with GC and combined it with the GEO database (five datasets GSE14210, GSE15459, GSE22377, GSE29272 and GSE51105 are combined, total number of cases = 592) for survival analysis to screen out the downregulated *LHPP* gene (Fig. 1A and Supplementary Fig. 1, 2). In the TCGA database, we found that the expression of LHPP in GC was significantly lower than that in normal tissues (Supplementary Fig. 3), which was consistent with our experimental results, and the expression of LHPP in GC tissues at the mRNA level (Fig. 1B) and protein level (Fig. 1C, D) were both lower than those in normal tissues. We further used IHC to evaluate the prognostic role of LHPP in GC. LHPP expression in normal tissues was significantly higher than that in gastric tumour tissues (Fig. 1E, F).

To further evaluate the value of LHPP in clinical prognosis and the benefit of adjuvant chemotherapy (ACT), survival analyses revealed that the overall survival rate of patients with low LHPP expression was significantly lower than that of patients with high LHPP expression (OS, 47.2 vs 63.0%, *P* = 0.005) (Fig. 1G). The further stratified analysis demonstrated that high expression of LHPP could significantly improve survival in patients receiving ACT (*P* = 0.009), whereas patients with low LHPP expression experienced no significant impact on survival, regardless of whether they received ACT (*P* = 0.092) (Fig. 1H). In addition, the same results were observed in the external validation set. Survival analysis showed that patients with high LHPP expression had significantly better overall survival than those with low LHPP expression (OS, 85.0 vs. 60.4%, *P* = 0.012) (Fig. 1I). Same as the internal set, whether patients with high LHPP expression received ACT had a significant difference in their survival rate (*P* = 0.022), while patients with low LHPP expression had no difference (*P* = 0.292) (Fig. 1J). Collectively, these results suggest that LHPP has potential clinical value as a predictive biomarker for disease outcomes in GC.

### LHPP suppressed the proliferation and metastasis of GC cells in vitro

**Fig. 2:**
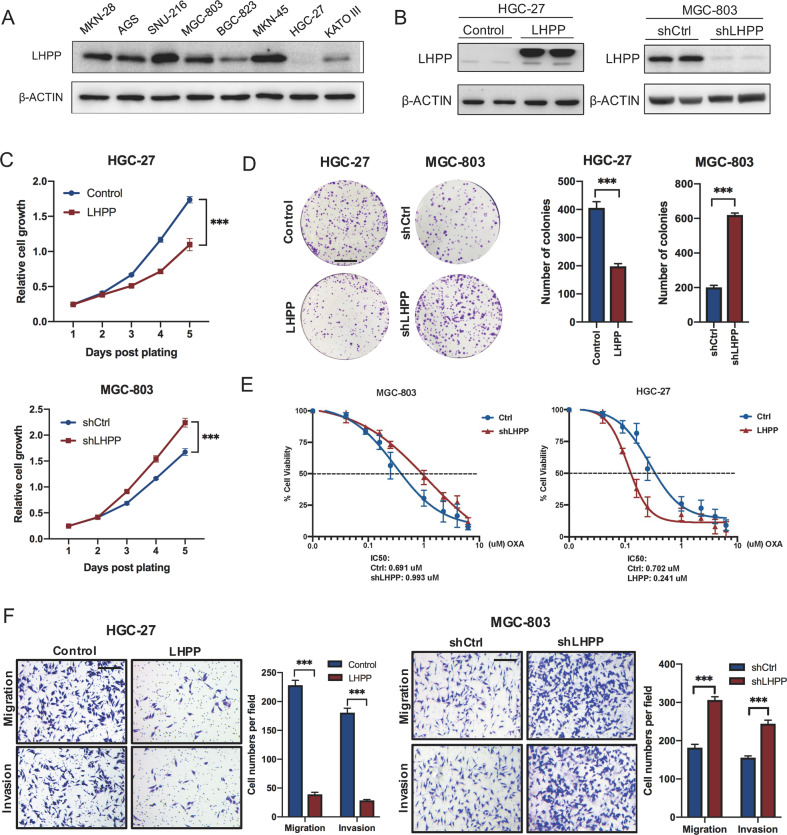
LHPP inhibits GC proliferation and metastasis in vitro. **A** Basic protein expression of LHPP in GC cell lines (MKN-28, AGS, SNU-216, MGC-803, BGC, MKN-45, HGC-27 and KATO III) was detected by western blotting. **B** HGC-27 cells with stable LHPP overexpression or MGC-803 cells with LHPP knockdown were created. The changes in LHPP expression were confirmed using western blotting. **C**, **D** The proliferative ability of stably transfected HGC-27 or MGC-803 cells was investigated via CCK-8 assays and colony formation. CCK-8 data were analyzed using a two-way analysis of variance. Colony number data were analyzed using the Wilcoxon test. Scale bars = 1 cm. **E** The drug resistance of stably transfected HGC-27 or MGC-803 cells was investigated via IC_50_ assays and colony formation. **F** Transwell assays with stably transfected HGC-27 and MGC-803 cells were performed. Representative images and quantification of the results are presented. Scale bars = 100 μm. Cell number data were analyzed using the Wilcoxon test. ^***^P < 0.001, GC gastric cancer, OXA Oxaliplatin.

To investigate the potential role of LHPP in the invasion and metastasis of GC, we detected the expression level of LHPP in several GC cell lines (Fig. 2A) and constructed HGC-27 and MGC-803 GC cell lines with stable overexpression or downregulation of LHPP (Fig. 2B). We found that knockdown of LHPP expression promoted the proliferation ability of MGC-803 cells in vitro; in contrast, upregulated LHPP expression levels resulted in a significant reduction in the proliferation ability (Fig. 2C, D). In addition, LHPP affected drug resistance. The plate clone and IC50 of the drug resistance experiment showed that the knockdown of LHPP increased the drug resistance of GC cells in vitro, whereas overexpression of LHPP caused a decrease in drug resistance of GC cells in vitro (Fig. 2E), which was verified in two cell lines (MGC-803 and MKN-28) (Supplementary Fig. 4). Furthermore, we also found that knockdown of LHPP expression promoted the migration and invasion abilities of cells in vitro; on the contrary, upregulated LHPP expression levels resulted in a significant decrease in migration and invasion abilities (Fig. 2F). Thus, these results demonstrated that LHPP suppressed GC tumorigenicity in vitro.

### LHPP suppressed the proliferation and metastasis of GC in vivo

**Fig. 3:**
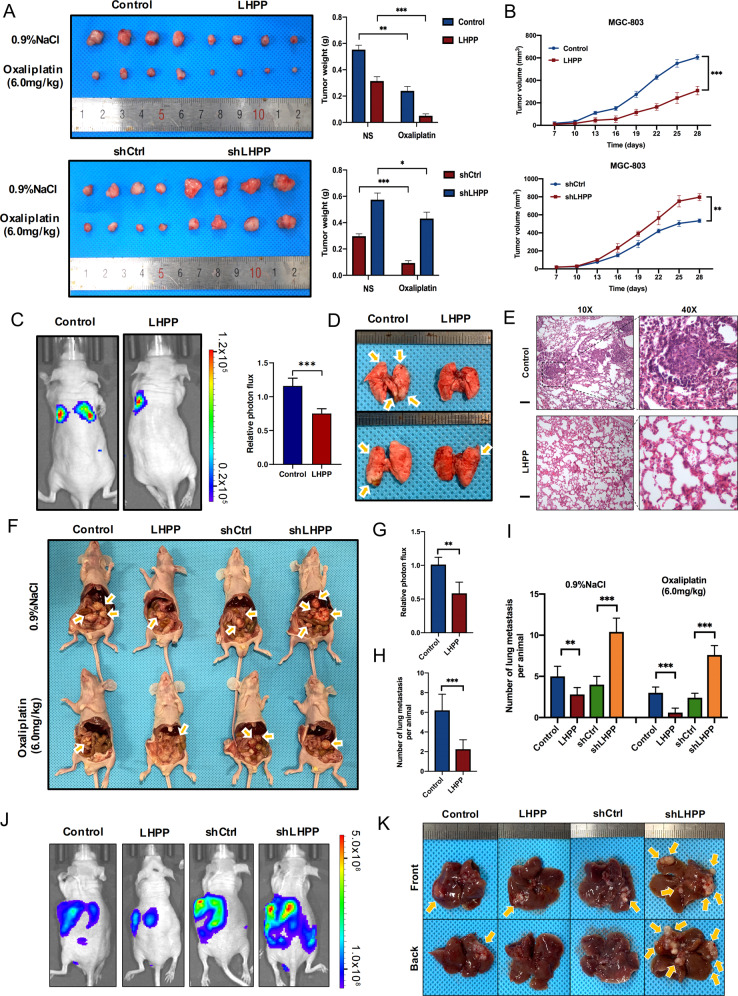
LHPP inhibits GC proliferation and metastasis in vivo. **A** Overexpression of LHPP inhibited GC growth in a subcutaneous xenograft model. LHPP knockdown promoted GC growth in a subcutaneous xenograft model. Tumours were extracted and weighed after mice were sacrificed (*n* = 4 per group). Tumour weight data were analyzed using the Wilcoxon test. **B** The size of the tumours was measured at the indicated time points. Data were analyzed using a two-way analysis of variance. Repeat measure ANOVA was performed to compare data involving multiple time points. **C** Representative bioluminescence images of mice at 4 weeks after tail vein injection of LHPP-overexpressing MGC-803 cells or control cells and quantification of the images (*n* = 5 per group). **D**, **E** Representative images of lung metastasis and hematoxylin-and-eosin staining. Metastatic nodules were counted with or without a microscope and recorded. Overexpression of LHPP in MGC-803 cells significantly reduced the number of metastatic lesions in the lungs. Scale bars = 200 μm. **F**–**I** Stably transfected MGC-803 cells were injected intraperitoneally, and the number of metastases in the colonic wall was recorded 4 weeks later. Peritoneal metastases were examined and recorded (*n* = 4 per group). Data were analyzed using the Wilcoxon test. **J** Representative bioluminescence images of mice at 4 weeks after spleen injection of LHPP-overexpressing or LHPP-knockdown MGC-803 cells or control cells and quantification of the images (*n* = 6 per group). **K** Representative images of liver metastasis. Metastatic nodules were counted with or without a microscope and recorded. Overexpression of LHPP in MGC-803 cells significantly reduced the number of metastatic lesions in the livers. Knockdown of LHPP in MGC-803 cells significantly increased the number of metastatic lesions in the livers. ^*^P < 0.05, ^**^P < 0.01, ^***^P < 0.001, GC gastric cancer.

To further verify the biological functions of LHPP in vivo, we constructed a subcutaneous tumour model in nude mice to evaluate the effect of LHPP on the tumorigenic ability of GC cells in vivo. After the overexpression of LHPP, tumour growth was inhibited, and the growth in tumour volume was significantly decelerated compared with that in the control group. After knocking down LHPP, the results were reversed. When establishing the nude mouse subcutaneous tumour model, we divided the nude mice into two groups: one group was injected with 0.9% NaCl, and the other group was treated with the chemotherapy drug Oxaliplatin. After 4 weeks, the cells overexpressing LHPP in the Oxaliplatin group showed stronger drug sensitivity than those in the control group, whereas the cells with knocked down LHPP showed stronger drug resistance than those in the control group (Fig. 3A, B). We further used IHC to consolidate LHPP expression in tumours (Supplementary Fig. 5).

To determine the role of LHPP in GC metastasis in vivo, MGC-803 cells overexpressing LHPP or corresponding control cells were injected into nude mice via the tail vein. When investigating the lungs 4 weeks after injection, mice injected with LHPP-overexpressing cells exhibited significantly reduced GC lung metastasis, as shown by bioluminescence imaging (Fig. 3C). The number of metastatic colonies in the control group was higher than that in the LHPP-overexpression group (Fig. 3D). Histological analysis of the dissected lungs using H&E staining confirmed that the control group had more metastatic nodules than the LHPP-overexpression group (Fig. 3E). Furthermore, enhanced LHPP expression significantly inhibited peritoneal metastasis after the intraperitoneal injection of GC cells. When establishing the tumour metastasis model by intraperitoneal injection, we also divided the nude mice into two groups: one group was injected with 0.9% NaCl, and the other group was treated with the chemotherapy drug Oxaliplatin. After 4 weeks, nude mice in the Oxaliplatin group injected with cells overexpressing LHPP had a much greater reduction in the number of metastatic nodules in the abdominal cavity than those in the control group, indicating that high LHPP expression can lead to stronger drug sensitivity; however, in the nude mice injected with LHPP knockdown cells, the degree of reduction of peritoneal metastasis nodules was not significantly different from that of the control group, indicating that LHPP knockdown would result in stronger drug resistance (Fig. 3F–I). In addition, we successfully established a liver-spleen metastasis model. The results revealed that the overexpression of LHPP can inhibit the liver metastasis of GC cells, whereas knocking down LHPP can promote liver metastasis of GC cells (Fig. 3J–K and Supplementary Fig. 6).

In summary, LHPP inhibited the proliferation, invasion, and metastasis of GC cells and reduced the drug resistance of GC cells in vivo.

### METTL14-mediated m6A modification of LHPP mRNA in GC

**Fig. 4:**
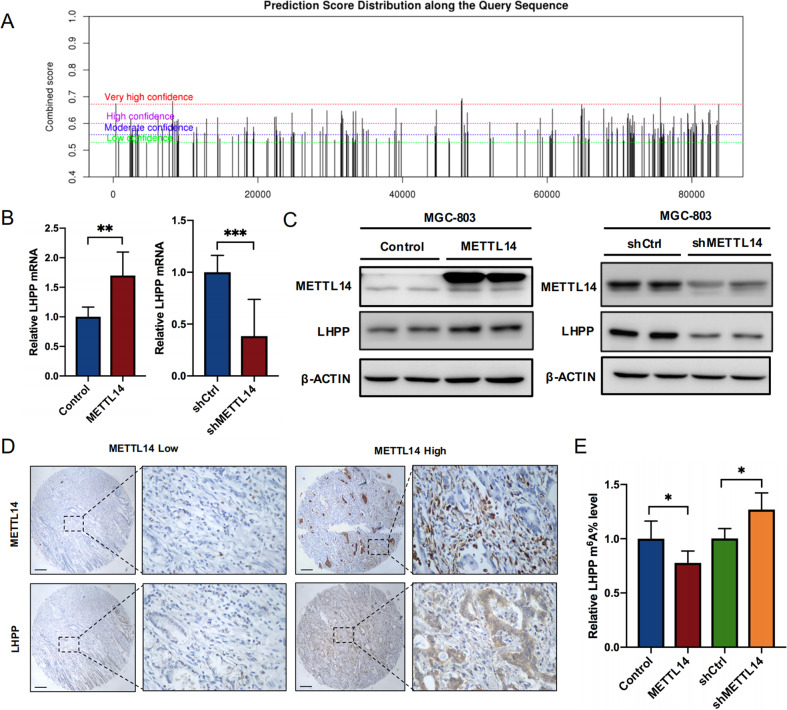
METTL14-mediated m6A modification represses LHPP expression in GC. **A** The potential m6A sites were predicted by SRAMP. **B**, **C** qRT-PCR and western blot assays revealed the mRNA and protein expression, respectively, of LHPP in GC cells with knockdown or overexpression of METTL14. **D** IHC staining of LHPP and METTL14 in TMAs. Scale bars = 200 μm. **E** m6A immunoprecipitation and qRT-PCR assays showed the relative percentage of LHPP mRNA with methylation. Data were analyzed using the Wilcoxon test. ^*^P < 0.05, ^**^P < 0.01, ^***^P < 0.001, ns no significant difference, GC gastric cancer, qRT-PCR quantitative reverse transcription-polymerase chain reaction, IHC immunohistochemical, TMS tissue microarrays.

The mechanisms leading to aberrant LHPP expression remain unclear. Considering the low mRNA level of LHPP in GC, we questioned whether m6A modification regulates the mRNA stability of LHPP. The online prediction tool SRAMP (http://www.cuilab.cn/sramp) shows that there are many m6A sites with high confidence in LHPP (Fig. 4A and Supplementary Fig. 7A). Furthermore, using the GEPIA database to analyse the correlation between many important m6A methyltransferases and demethyltransferases and LHPP, we found that the methyltransferase METTL14 had a significantly positive correlation with LHPP (Supplementary Fig. 7B). Therefore, we knocked down the expression of METTL14 and found that the expression of LHPP also decreased, and vice versa (Fig. 4B, C). In addition, TMA-based IHC revealed that the expression of METTL14 in GC was positively correlated with LHPP (Fig. 4D). As shown in Fig. 4E, we found that m6A modification of LHPP mRNA was positively correlated with the expression of METTL14 in GC cells. These results proved that the mRNA stability of LHPP was regulated by METTL14-mediated m6A methylation.

### LHPP is modified by acetylation to exert a tumour suppressor function

**Fig. 5:**
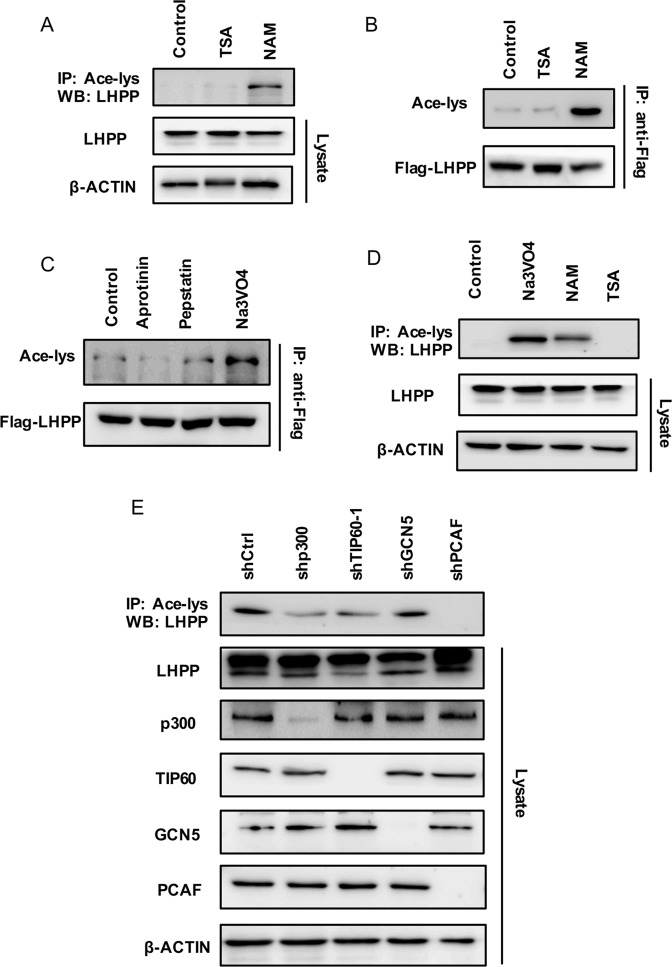
LHPP is modified by acetylation to exert a tumour suppressor function. **A** Acetylation of exogenous Flag-LHPP in MGC-803 cells treated with deacetylase inhibitors, TSA or NAM. Flag-LHPP was immunoprecipitated with anti-Flag, and the precipitates were analysed using an anti-acetyl-lys antibody (Ace-lys). **B** Acetylation of endogenous LHPP in MGC-803 cells treated with TSA or NAM. LHPP acetylation was analysed using immunoprecipitation with an anti-acetyl-lys antibody followed by western blotting for LHPP. **C** Acetylation of Flag-LHPP in MGC-803 cells treated with phosphatase inhibitors aprotinin, pepstatin and Na_3_VO_4_. Flag-LHPP was immunoprecipitated with anti-Flag and analysed by western blot using anti-phospho-serine. **D** Na_3_VO_4_ induced the acetylation of endogenous LHPP in MGC-803 cells on western blot. **E** Acetylation of LHPP in MGC-803 cells infected with lentivirus expressing each of the indicated acetyltransferase shRNAs. TSA trichostatin A, NAM nicotinamide, shRNA short hairpin RNA.

To explore how LHPP molecules exert tumour biological functions, we performed an enrichment analysis of the protein modification functions of KEGG and GO (Supplementary Fig. 8). The results demonstrated that LHPP was highly related to acetylation modification, and there might be acetylation sites (Supplementary Figs. 8, 9). To test whether LHPP is an acetylated protein, we analysed the acetylation of endogenous LHPP (Fig. 5A) and exogenously expressed LHPP (Fig. 5B) in cells treated with trichostatin A (TSA), a broad-spectrum inhibitor of the histone deacetylases (HDAC) family of deacetylases, and nicotinamide (NAM), an inhibitor of the sirtuins (SIRT) family of deacetylases. Using a specific antibody against acetylated lysine, we detected strong acetylation of both exogenous and endogenous LHPP in NAM-treated but not TSA-treated cells (Fig. 5A, B). To clarify which phosphatase inhibitor mediates the acetylation of LHPP, we performed exogenous and endogenous acetylation of LHPP cells treated with three phosphatase inhibitors, aprotinin, pepstatin and Na_3_VO_4_. Surprisingly, we found that Na_3_VO_4_ stimulated the acetylation of exogenous and endogenous LHPP (Fig. 5C, D). These results suggest that LHPP is an acetylated protein and that acetylation can be specifically triggered by the phosphatase inhibitor Na_3_VO_4_. To determine the acetyltransferase which regulates LHPP acetylation, we knocked down the acetyltransferases p300, TIP60, GCN5 and PCAF in cells overexpressing LHPP. The results revealed that the knockdown of PCAF, but not the other acetyltransferase genes, largely reduced the basal acetylation level of LHPP (Fig. 5E). The above results indicated that LHPP is an acetylated protein regulated by phosphatase inhibitors, and its acetyltransferase is the PCAF of the Kats family. Thus, LHPP exerts a tumour suppressor effect through acetylation.

### LHPP suppressed aerobic glycolysis

**Fig. 6:**
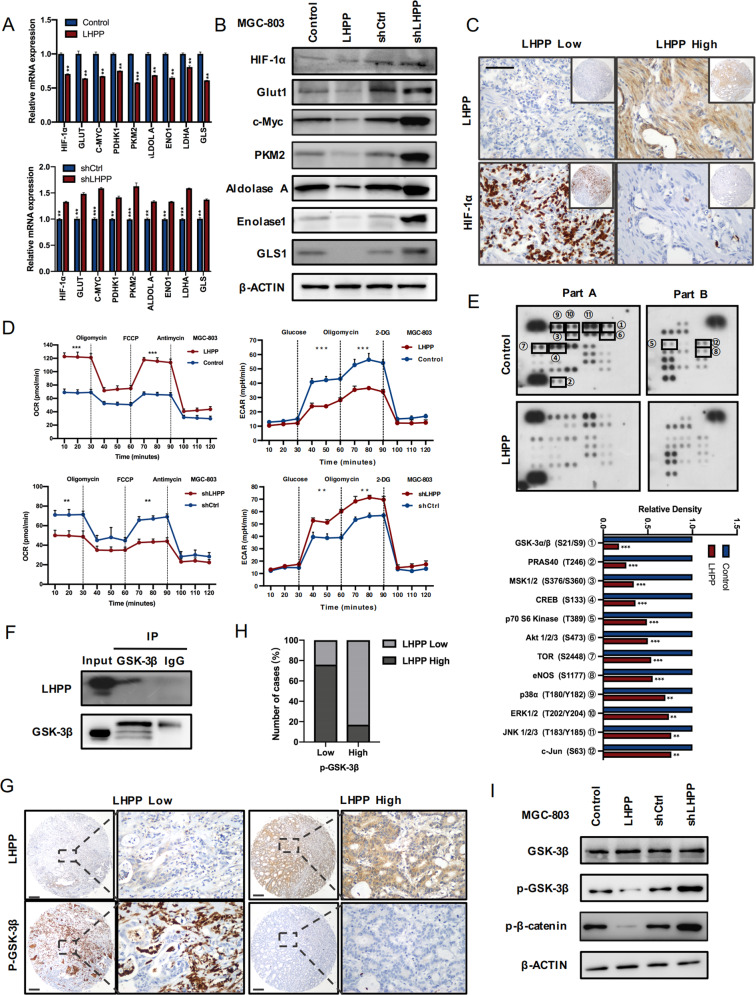
LHPP suppresses aerobic glycolysis. **A** Relative mRNA expression levels of glycolytic genes and glutamine transporters in LHPP-overexpressing or LHPP-knockdown MGC-803 cells and control cells by qPCR. **B** Protein expression levels of glycolytic genes and glutamine transporters in LHPP-overexpressing or LHPP-knockdown MGC-803 cells and control cells on western blot. **C** IHC staining of LHPP and HIF1A in TMAs and their correlation. Scale bars = 100 μm. **D** Oxygen consumption rate and extracellular acidification rate of LHPP-overexpressing or LHPP-knockdown MGC-803 cells and control cells were measured using the Seahorse Bioscience XF96 analyser. **E** Human phosphokinase microarray assay analysis of the conditioned medium from stably transfected HGC-27 cells. A summary of the relative signal intensities of the indicated proteins is shown. **G**, **H** IHC staining of LHPP and p-GSK3b in TMAs and their correlation. Scale bars = 200 μm. **F** Combined LHPP and GSK3b by co-immunoprecipitation. **I** Protein expression levels of the Wnt pathway in LHPP-overexpressing or LHPP-knockdown MGC-803 cells and control cells on western blot. Data were analyzed using the Wilcoxon test. *P < 0.05, ^**^P < 0.01, ^***^P < 0.001, KEGG Kyoto encyclopaedia of genes and genomes, GO gene ontology, qPCR quantitative polymerase chain reaction, IHC immunohistochemical, TMA tissue microarray.

To explore how LHPP affects the proliferation and invasion of GC, we conducted an enrichment analysis of the Reactome pathway and the biological functions of LHPP. The results revealed that LHPP is significantly related to the Akt signalling pathway, Wnt signalling pathway, and cell energy metabolism pathways (Supplementary Figs. 10–12). The downstream effectors of the PI3K/Akt and Wnt pathways play a central role in cancer cell metabolic reprogramming. For example, the hyperactivation of Akt promotes aerobic glycolysis and glutaminolysis, and c-Myc activates the transcription of various glycolytic and glutaminolysis genes. Therefore, we detected the expression levels of glycolysis-related proteins HIF1A, GLUT1, C-MYC, PDHK1, PKM2, ALDOL A, ENO1, LDHA and GLS in MGC-803 cells overexpressing or with knocked down LHPP (Fig. 6A, B). The results revealed that LHPP could inhibit glycolysis-related proteins, thereby inhibiting aerobic glycolysis in GC cells. In further immunohistochemical testing, we found that LHPP and hypoxia-inducible factor HIF1A were significantly negatively correlated (Fig. 6C). In the experiment on cell oxygen consumption rate and extracellular acidification rate, overexpression/knockdown of LHPP respectively increased or decreased the cell oxygen consumption rate and decreased or increased the extracellular acidification rate (Fig. 6D and Supplementary Fig. 13). These results showed that LHPP could inhibit aerobic glycolysis in GC cells.

To further clarify the pathways through which LHPP affects glycolysis, we used the Human PhosphoKinase Array Kit to identify phosphorylated kinases that are closely related to LHPP. The results demonstrated that the expression levels of multiple phosphorylated protein kinases were different between the control and LHPP-overexpressing GC HGC-27 cells, among which phosphorylated GSK-3β and Akt levels changed most significantly (Fig. 6E). IHC and western blot experiments also proved that LHPP was negatively correlated with the phosphorylation of GSK-3β, and LHPP could also regulate the phosphorylation of β-catenin, a key molecule in the Wnt pathway (Fig. 6G–I). In addition, through the STRING database and endogenous co-immunoprecipitation experiments, we further proved that LHPP and GSK-3β interacted (Fig. 6F and Supplementary Fig. 13E). Further rescue experiments confirmed previous results (Supplementary Fig. 14). In summary, LHPP inhibited the Wnt pathway by inhibiting the phosphorylation of GSK-3β, thereby inhibiting the growth of GC cells.

## Discussion

A key factor leading to the occurrence of aerobic glycolysis in tumour cells is that hypoxia or oncogene expression leads to the activation of hypoxia-inducible factor-1 (HIF-1) [19, 20]. HIF-1 is a heterodimeric protein composed of a stable b subunit and an unstable gene. Under positive oxygen pressure, oxygen-dependent hydroxyproline dioxygenase is linked to VHL. With the expression of the enzyme, the synthesised HIF-1 is subsequently degraded by VHL ubiquitination. Under hypoxic stress, HIF-1 promotes the conversion of glucose to acetone by upregulating the expression of Glut1, HK1, HK2, lactate dehydrogenase A (LDH-A) and MCT4 (monocarboxylate transporter 4), and the aerobic glycolysis of acid and lactic acid proceeds [21]. In addition, HIF-1 also activates the expression of PDK1 (pyruvate dehydrogenase kinase) through transcription while inhibiting pyruvate dehydrogenase (PDH) to convert pyruvate into acetyl-coenzyme A (Ac-CoA), while CoA is normal, the raw materials which enter the tricarboxylic acid cycle and the electron transport provider in respiratory chain complexes 1 and 2 [22]. Therefore, by inhibiting PDH, HIF-1 restricts the progress of mitochondrial oxidative phosphorylation. In addition, HIF-1 cooperates with c-Myc to induce the expression of HK2 and PDK1, thereby further promoting the progress of aerobic glycolysis, enabling tumour cell protein synthesis, cell cycle progression and metabolic reprogramming, and finely regulating tumour cell growth and the metabolic adaptive response in a hypoxic environment [23–25].

The regulation of glucose metabolism genes by transcription factors is an important mechanism in tumour energy metabolism reprogramming [26]. In addition to the aforementioned HIF-1α, transcription factor FOXO1, signal transducer and activator of transcription 3 (STAT3), Sp1, etc., mainly act as tumour suppressor genes in tumours [27]. Demaria et al. found that under stress conditions, tyrosine 705 is activated by phosphorylation, and transcription activates HIF-1α to promote glycolysis [28]. The specificity protein (Sp) is a member of the Sp/Kruppel-like factor family. Sp1 is overexpressed in some tumours and often interacts with the PI3K/AKT signalling pathway and other transcription factors to regulate tumour glycolysis. Kao et al. reported that endothelin-1 (ET-1) and cAMP can synergistically activate Sp1 to promote GLUT1 transcription, thereby regulating the glycolysis process in tumour cells [29].

The specific molecular mechanism of the high glycolysis energy metabolism phenotype of tumour cells has not yet been fully elucidated. Recent studies have shown that the regulation of aerobic oxidation is related to abnormalities in multiple signalling pathways [30]. In addition, the opening and closing of the Wnt signalling pathway directly control the expression levels of a large number of genes related to growth and metabolism. Pate et al. found that Wnt signalling can regulate glycolysis and angiogenesis through PDK1 and interfere with colon cancer cells. Wnt signalling can reduce glycolytic metabolism and inhibit the growth of tumour cells [31]. Preliminary research by the project team also found that in GC cells, Akt can mediate the phosphorylation of GSK-3β, which in turn affects the expression of the β-catenin protein, thereby regulating GC invasion and metastasis [32]. Many tyrosine kinase receptors, such as epidermal growth factor receptor (EGFR) and platelet-derived growth factor receptor (PDGFR), can activate PI3K on the cell surface; subsequently, Akt is recruited to the cell and activates mTOR, which can induce HIF-1 to promote glycolysis in cells [33].

**Fig. 7:**
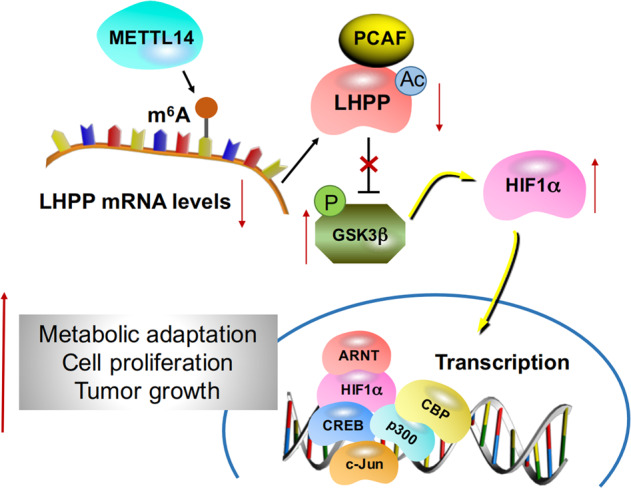
Proposed mechanism scheme of LHPP in GC. The m6A modification of LHPP mRNA by METTL14 represses its expression; LHPP inhibits the phosphorylation of GSK3b through acetylation and mediates HIF1A to inhibit glycolysis, proliferation, invasion and metastasis of gastric cancer cells.

In conclusion, we found that LHPP acts as a tumour suppressor in GC by inhibiting cell proliferation, invasion, and drug resistance. We further proposed that LHPP is regulated by m6A methylation and regulates the metabolism of GC by changing the acetylation level. Thus, LHPP is a potential predictive biomarker and therapeutic target for GC (Fig. 7). The study of the influence of LHPP on the metabolic Warburg effect and biological phenotype of GC and its specific mechanisms will further deepen the understanding of the metabolic adaptation, occurrence, and development of GC cells and provide new candidate targets and intervention methods for the treatment of GC.

## Conclusions

LHPP is regulated by m6A methylation and regulates the metabolism of GC by changing the acetylation level. Thus, LHPP is a potential predictive biomarker and therapeutic target for GC.

## Supplementary information

Original Western Blots

Supplementary figure legends

A reproducibility checklist that details key elements of the experimental and analytical design of the submission.

Supplementary Figure 1

Supplementary Figure 2

Supplementary Figure 3

Supplementary Figure 4

Supplementary Figure 5

Supplementary Figure 6

Supplementary Figure 7

Supplementary Figure 8

Supplementary Figure 9

Supplementary Figure 10

Supplementary Figure 11

Supplementary Figure 12

Supplementary Figure 13

Supplementary Figure 14

## Data Availability

All data generated or analyzed during this study are included in this published article and its Supplementary Information file.
