# Supplementary material for: m6A methylation mediates LHPP acetylation as a tumour aerobic glycolysis suppressor to improve the prognosis of gastric cancer
Source: Cell Death Dis. 2022 May 14;13(5):463. doi: 10.1038/s41419-022-04859-w (PMC9107493; doi:10.1038/s41419-022-04859-w)

Figure1

D.

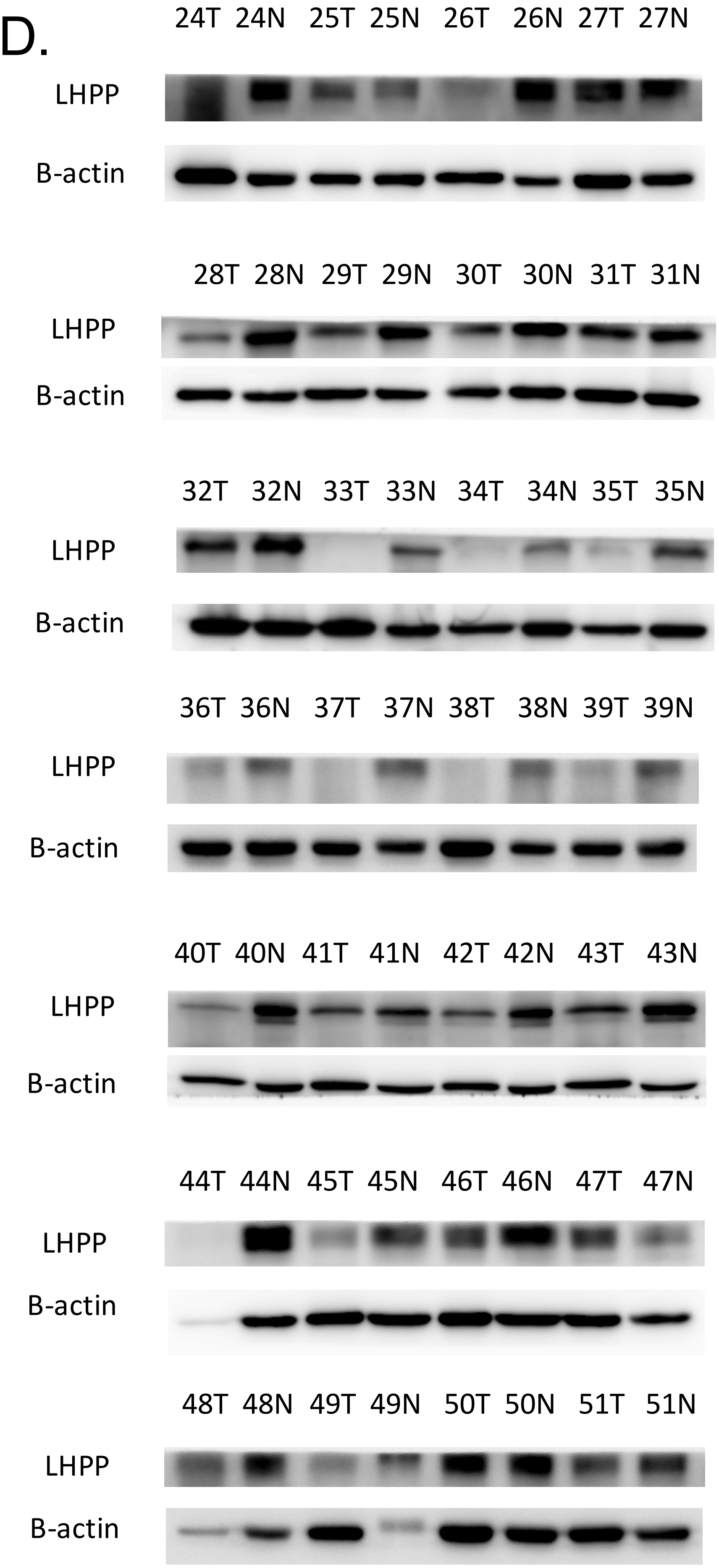
24T 24N 25T 25N 26T 26N 27T 27N
LHPP
B-actin
28T 28N 29T 29N 30T 30N 31T 31N
LHPP
B-actin
32T 32N 33T 33N 34T 34N 35T 35N
LHPP
B-actin
36T 36N 37T 37N 38T 38N 39T 39N
LHPP
B-actin
40T 40N 41T 41N 42T 42N 43T 43N
LHPP
B-actin
44T 44N 45T 45N 46T 46N 47T 47N
LHPP
B-actin
48T 48N 49T 49N 50T 50N 51T 51N
LHPP
B-actin

Figure1

D.

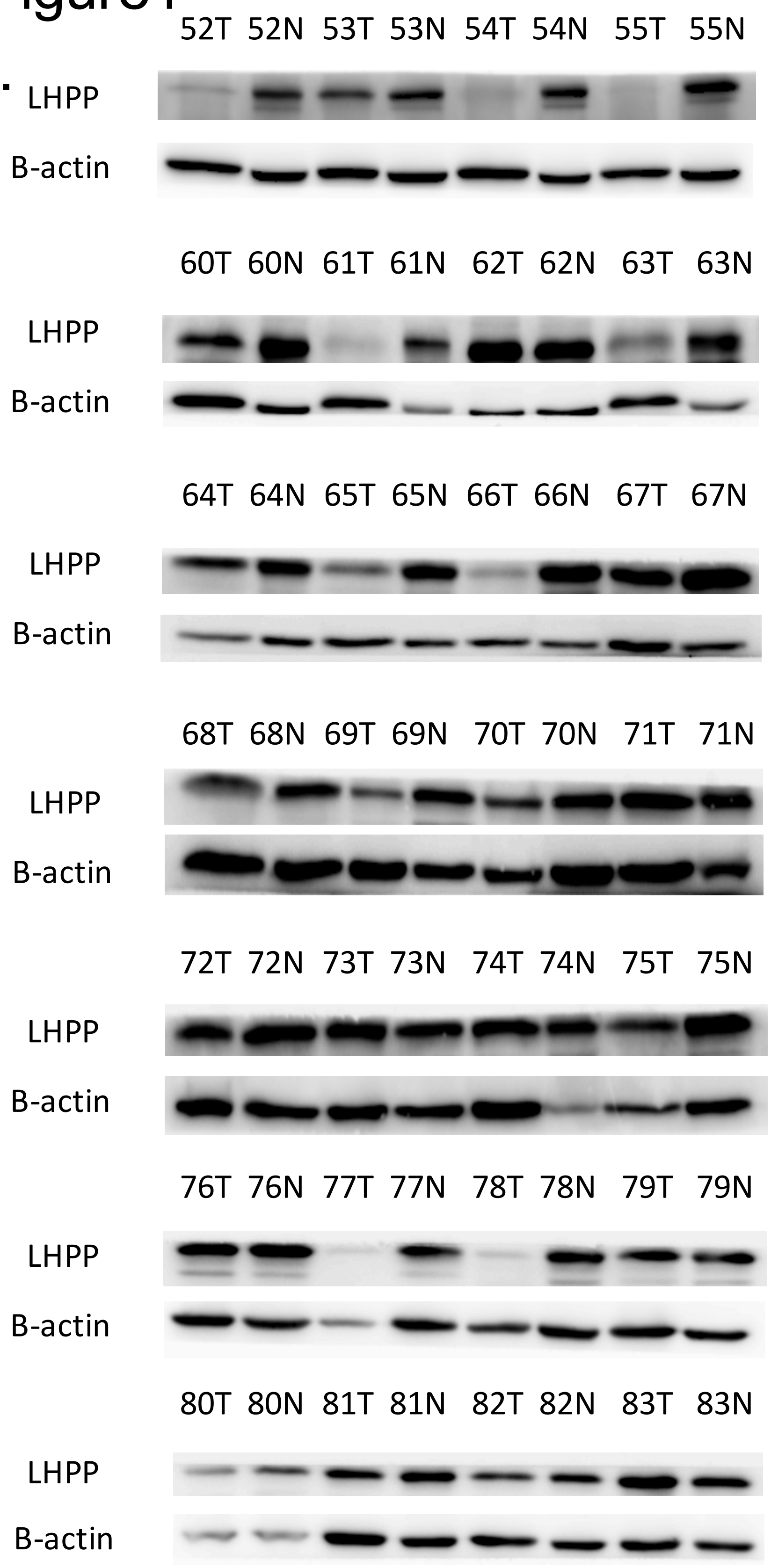
52T 52N 53T 53N 54T 54N 55T 55N
LHPP
B-actin
60T 60N 61T 61N 62T 62N 63T 63N
LHPP
B-actin
64T 64N 65T 65N 66T 66N 67T 67N
LHPP
B-actin
68T 68N 69T 69N 70T 70N 71T 71N
LHPP
B-actin
72T 72N 73T 73N 74T 74N 75T 75N
LHPP
B-actin
76T 76N 77T 77N 78T 78N 79T 79N
LHPP
B-actin
80T 80N 81T 81N 82T 82N 83T 83N
LHPP
B-actin

Figure1

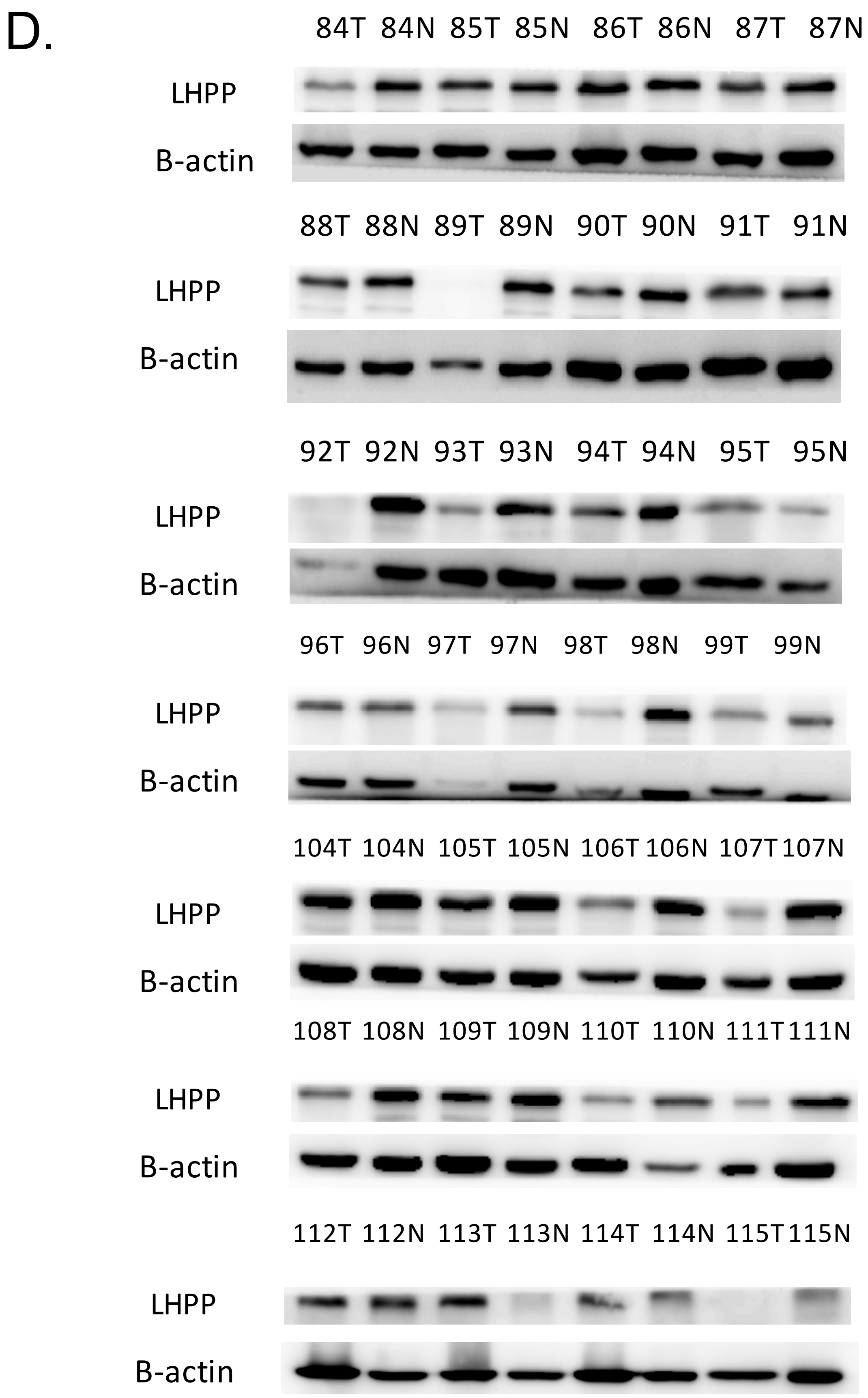
D.
84T 84N 85T 85N 86T 86N 87T 87N
LHPP
B-actin
88T 88N 89T 89N 90T 90N 91T 91N
LHPP
B-actin
92T 92N 93T 93N 94T 94N 95T 95N
LHPP
B-actin
96T 96N 97T 97N 98T 98N 99T 99N
LHPP
B-actin
104T 104N 105T 105N 106T 106N 107T 107N
LHPP
B-actin
108T 108N 109T 109N 110T 110N 111T 111N
LHPP
B-actin
112T 112N 113T 113N 114T 114N 115T 115N
LHPP
B-actin

Figure1

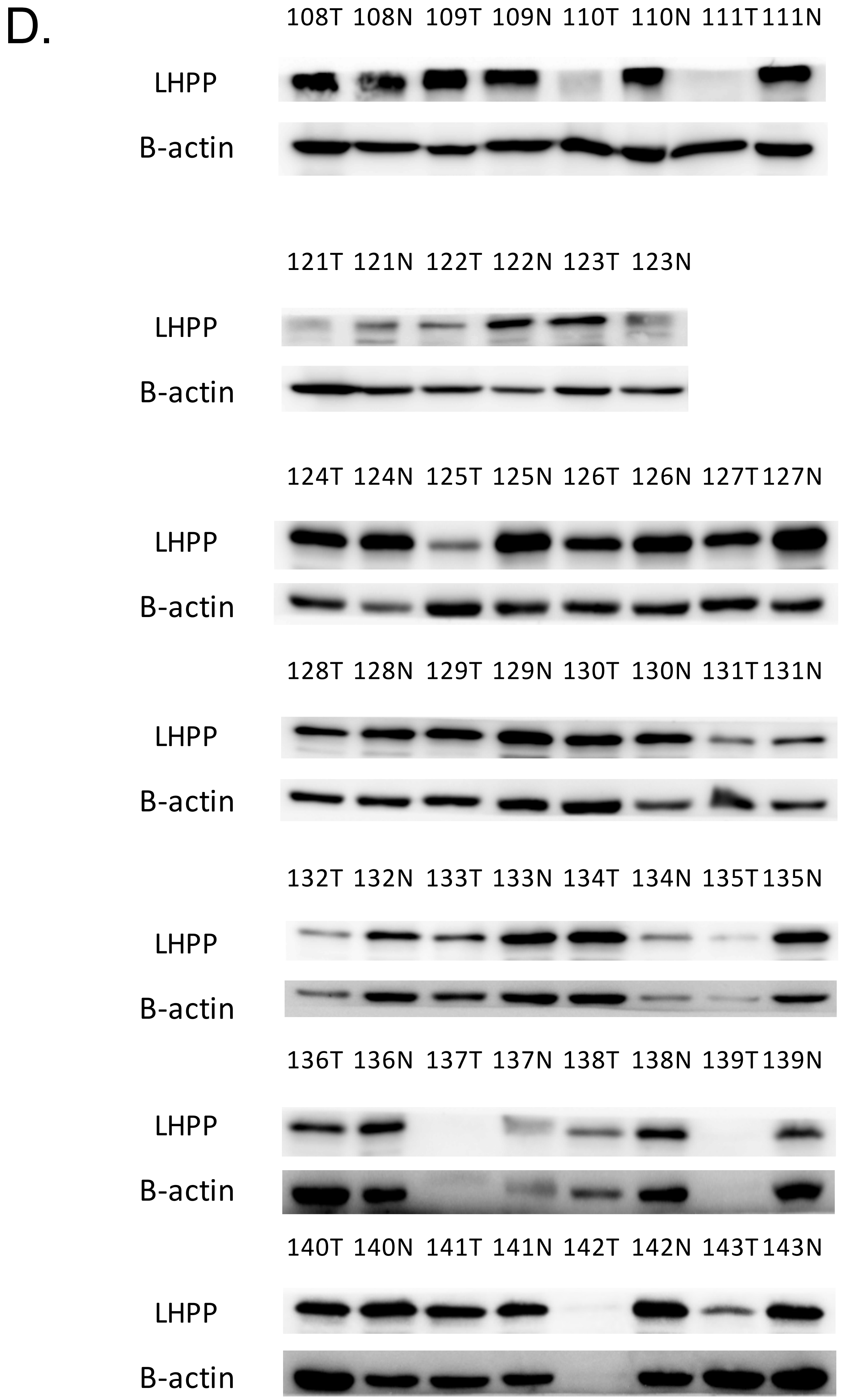
D.
108T 108N 109T 109N 110T 110N 111T 111N
LHPP
B-actin
121T 121N 122T 122N 123T 123N
LHPP
B-actin
124T 124N 125T 125N 126T 126N 127T 127N
LHPP
B-actin
128T 128N 129T 129N 130T 130N 131T 131N
LHPP
B-actin
132T 132N 133T 133N 134T 134N 135T 135N
LHPP
B-actin
136T 136N 137T 137N 138T 138N 139T 139N
LHPP
B-actin
140T 140N 141T 141N 142T 142N 143T 143N
LHPP
B-actin

Figure1

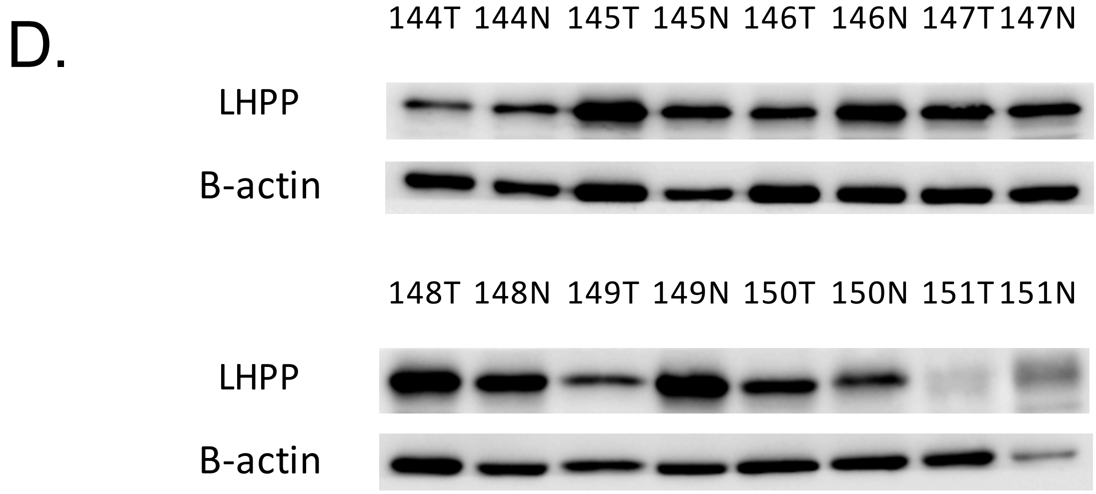

Figure2

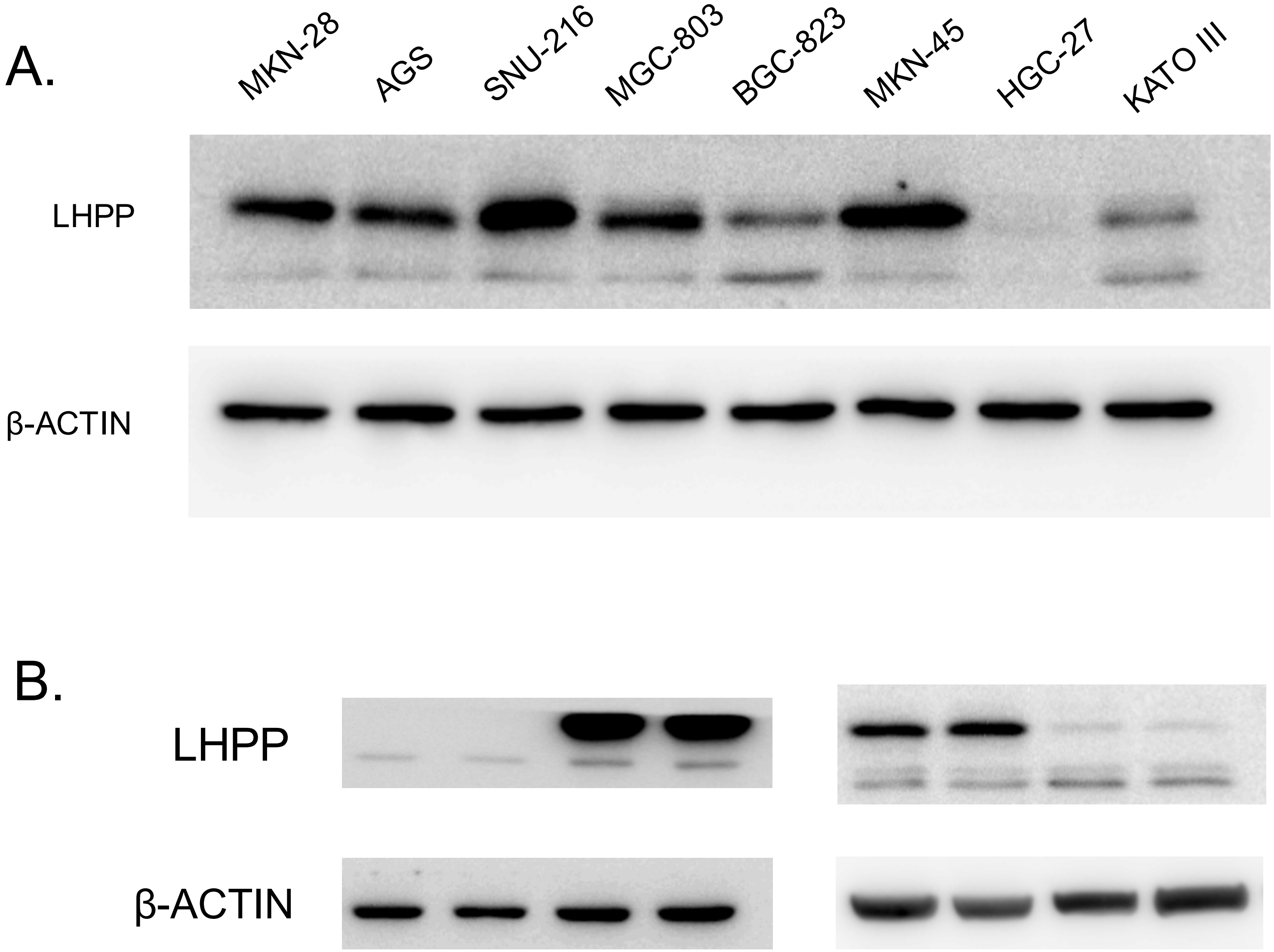

Figure4

C.

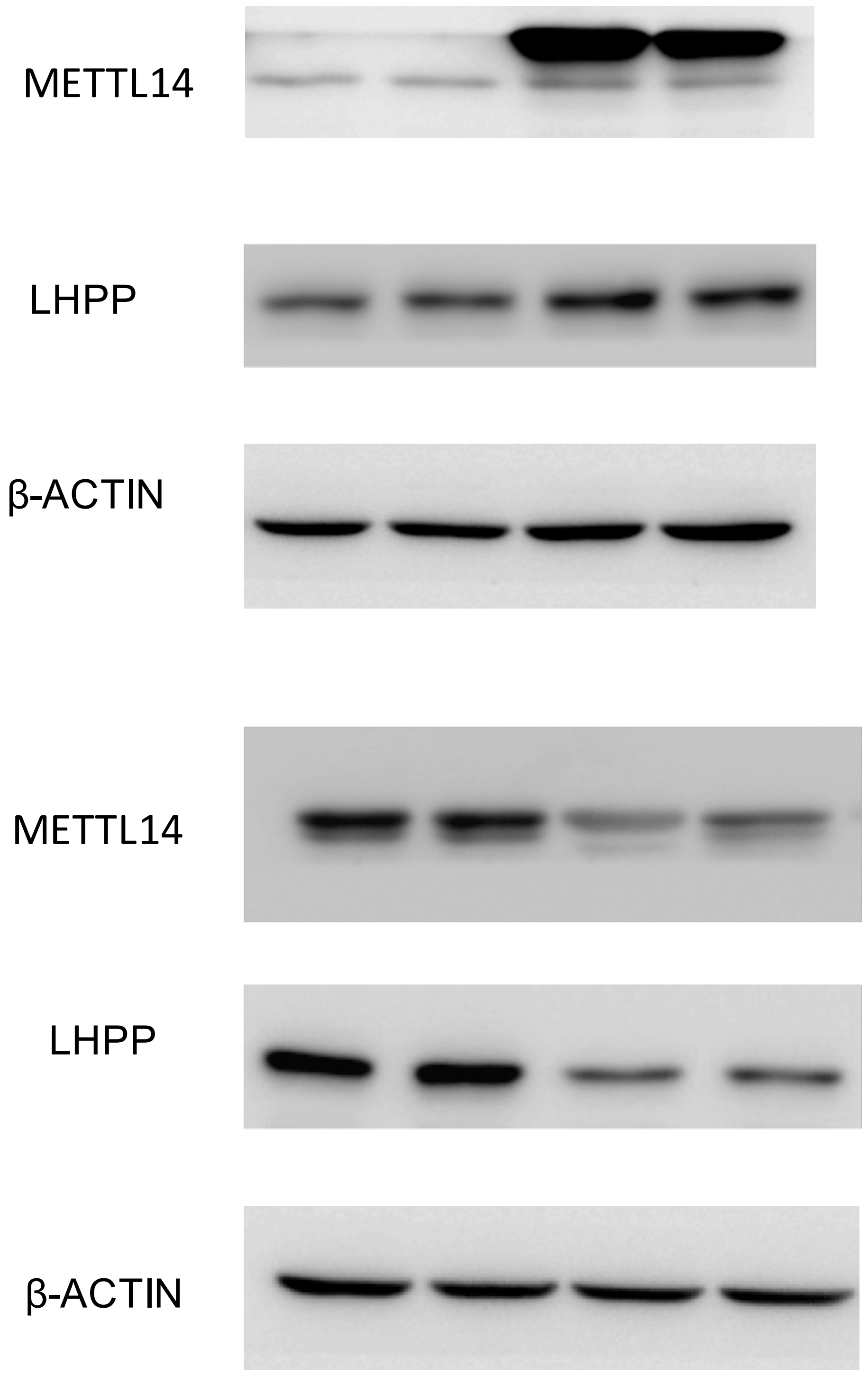

Figure5

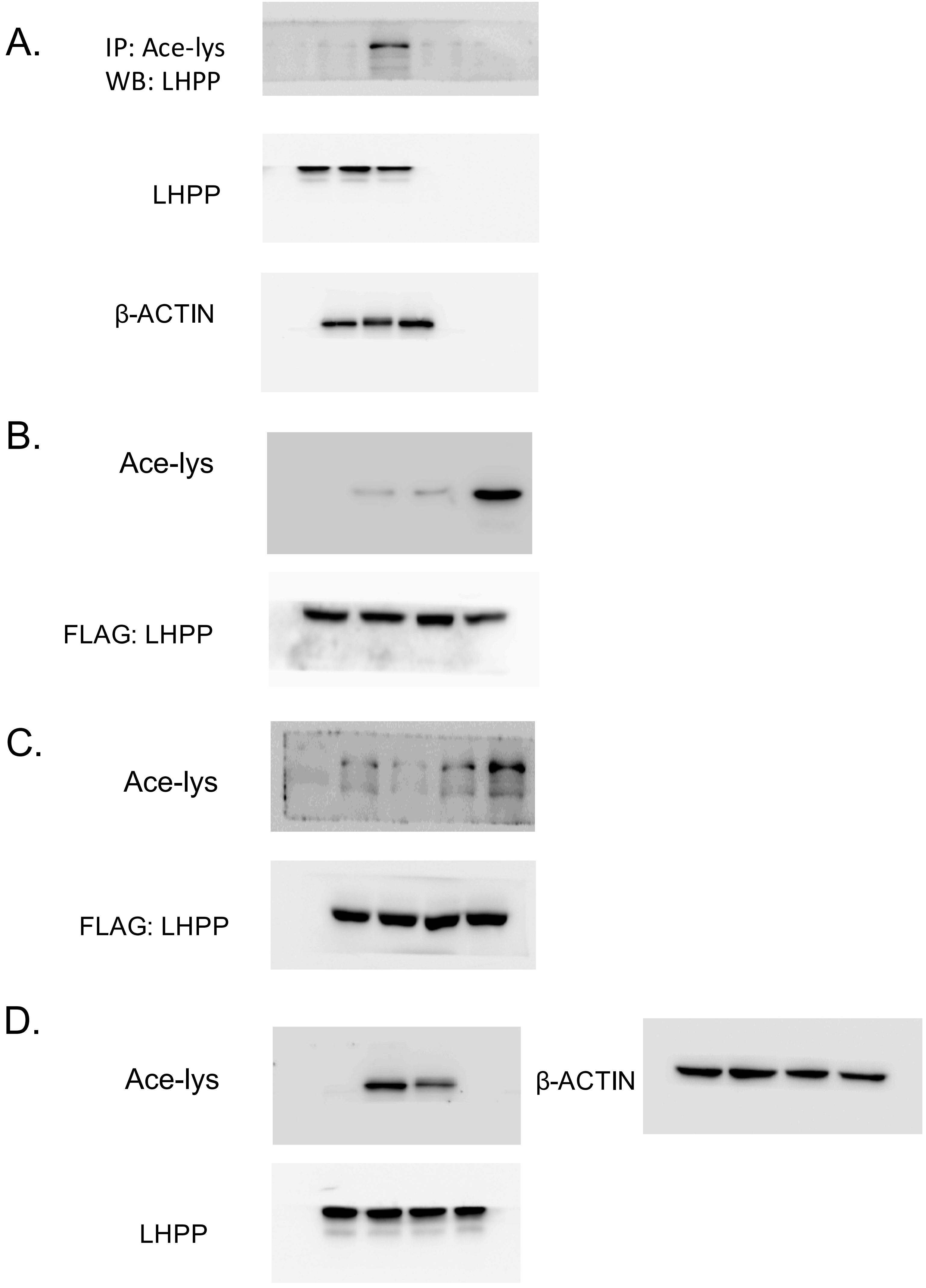
A.
IP: Ace-lys
WB: LHPP
LHPP
β-ACTIN
B.
Ace-lys
FLAG: LHPP
C.
Ace-lys
FLAG: LHPP
D.
Ace-lys
β-ACTIN
LHPP

E.

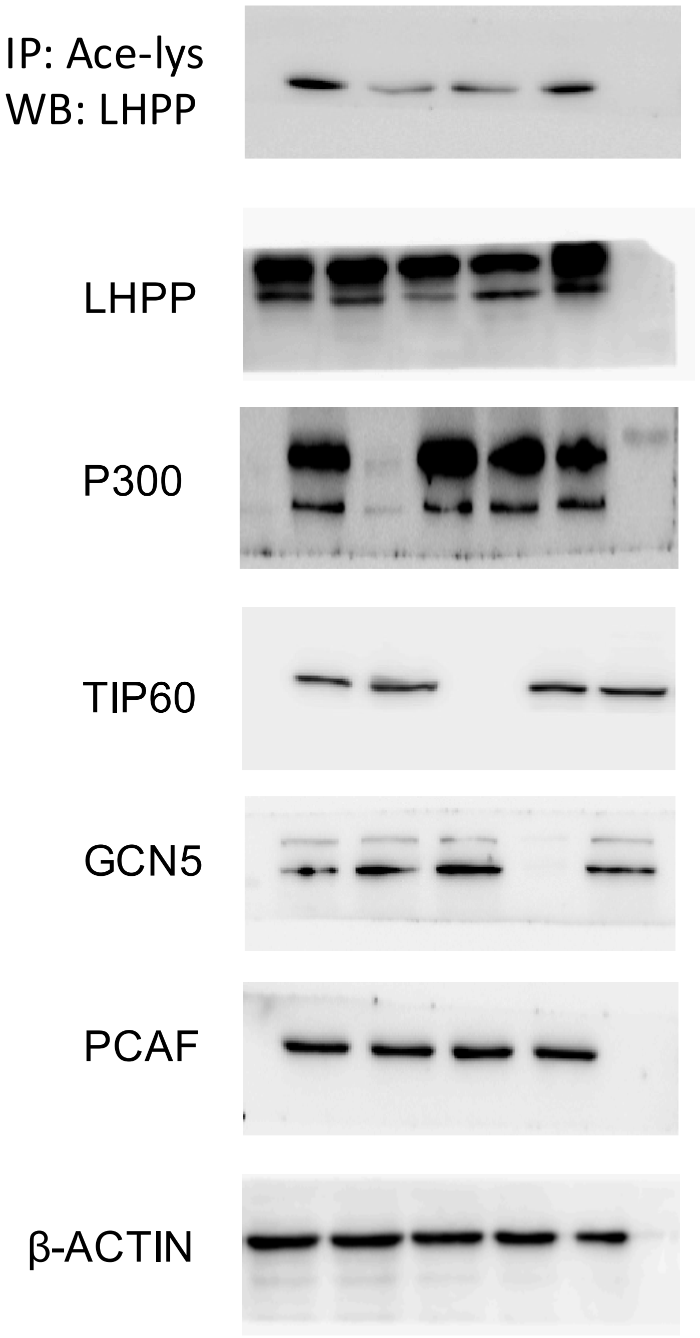

Figure6

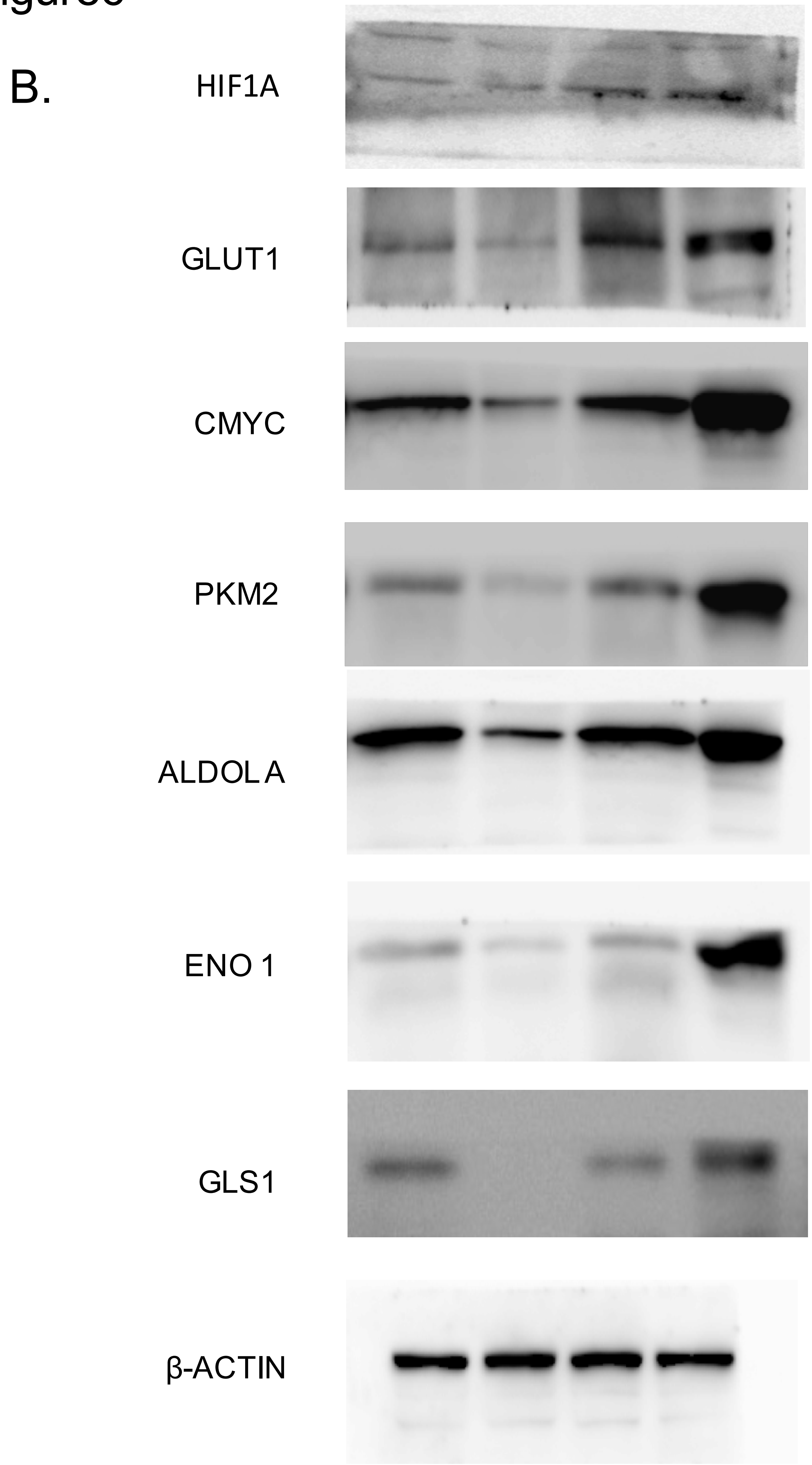

Figure6

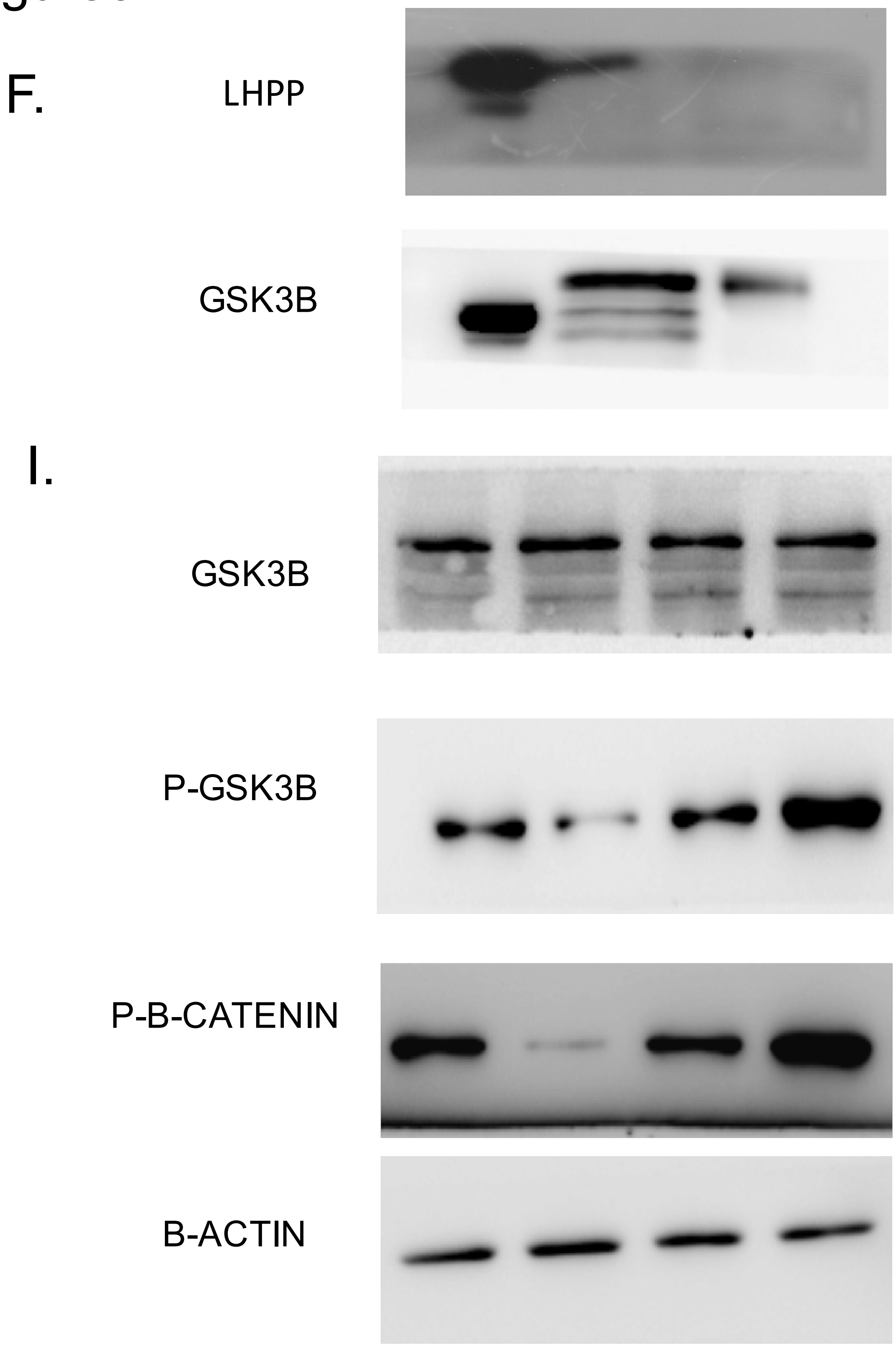

Supplement: Supplementary file 1 — Original Western Blots [file 41419_2022_4859_MOESM1_ESM.pdf]
