# Supplementary material for: m6A methylation mediates LHPP acetylation as a tumour aerobic glycolysis suppressor to improve the prognosis of gastric cancer
Source: Cell Death Dis. 2022 May 14;13(5):463. doi: 10.1038/s41419-022-04859-w (PMC9107493; doi:10.1038/s41419-022-04859-w)
Supplement: Supplementary file 2 — Supplementary figure legends [file 41419_2022_4859_MOESM2_ESM.docx]

**Supplementary figure legends**

**Supplementary Figure 1** (A-C) Volcano map of the differential genes according to the sequencing results of our centre. (D) Venn diagram of the differential genes according to the sequencing results of our centre. (E) Kaplan-Meier analyses of the correlations between LHPP expression and overall survival in the GEO_218523 cohort. (F) Kaplan-Meier analyses of the correlations between LHPP expression and overall survival in the TCGA database.

TGCA, The Cancer Genome Atlas

**Supplementary Figure 2** Survival analysis of LHPP, SPIRE1, SORBS1, L3MBTL3, NAPB, ARHGAP24 and CHD9. *P*-values for all survival analyses were calculated using the log-rank test.

**Supplementary Figure 3** Difference in LHPP protein expression between gastric tumours and adjacent normal gastric tissues in the TCGA database.

TGCA, The Cancer Genome Atlas

**Supplementary Figure 4** (A) The drug resistance of stably transfected MGC-803 cells was investigated colony formation. (B) MKN-28 cells with stable LHPP overexpression or LHPP knockdown were created. The changes in LHPP expression were confirmed using western blotting. (C) The drug resistance of stably transfected MKN-28 cells was investigated via IC_50_ assays.

**Supplementary Figure 5** Immunohistochemistry was used to visualize the LHPP expression in tumors. Scale bars = 100 μm.

**Supplementary Figure 6** (A) Representative images of liver metastasis and hematoxylin-and-eosin staining. Scale bars = 200 μm

**Supplementary Figure 7** (A) Potential m6A sites predicted by the SYSU database. (B) Correlation of LHPP and METTL13, METTL14, METTL16, FMR1, NSUN2, WTAP, YTHDF2, YTHDF3 according to the GEPIA database.

SYSU, Sun Yat-sen University; GEPIA, Gene Expression Profiling Interactive Analysis.

**Supplementary Figure 8** Enrichment analysis of LHPP protein modification functions of KEGG.

KEGG, Kyoto Encyclopedia of Genes and Genomes.

**Supplementary Figure 9** Enrichment analysis of LHPP protein modification functions of GO.

GO, Gene Ontology.

**Supplementary Figure 10** (A, B) Binding of energy metabolism-related molecules to LHPP according to the analysis of the STRING database. (C) Enrichment analysis of LHPP protein-related pathways according to the sequencing results of our centre. (D, E) Enrichment analysis of LHPP protein functions and related pathways according to KEGG and GO.

KEGG, Kyoto Encyclopedia of Genes and Genomes; GO, Gene Ontology

**Supplementary Figure 11** Enrichment analysis of LHPP protein functions of KEGG.

KEGG, Kyoto Encyclopedia of Genes and Genomes.

**Supplementary Figure 12** Enrichment analysis of LHPP protein functions of GO.

GO, Gene Ontology.

**Supplementary Figure 13** (A-D) Oxygen consumption rate and extracellular acidification rate of LHPP-overexpressing or LHPP-knockdown MKN-28 cells and control cells measured using the Seahorse Bioscience XF96 analyser. (E) Combination of LHPP and GSK3b according to the STRING database.

**Supplementary Figure 14** (A, B) The effect of LHPP overexpression on HGC-27 cell migration and invasion was rescued by transfection with overexpression HIF1a. (C, D) The effect of LHPP knockdown on MKN-28 cell migration and invasion was rescued by transfection with siHIF1a or 2-DG. (E) The effect of LHPP overexpression on HGC-27 cell proliferation was rescued by transfection with overexpression HIF1a. The effect of LHPP knockdown on MKN-28 cell proliferation was rescued by transfection with siHIF1a or 2-DG.

^**^P<0.01, ^***^P<0.001

ns, no significant difference
